# Use of a highly sensitive strand-specific quantitative PCR to identify abortive replication in the mouse model of respiratory syncytial virus disease

**DOI:** 10.1186/1743-422X-7-250

**Published:** 2010-09-22

**Authors:** Richard Bannister, Deborah Rodrigues, Edward J Murray, Carl Laxton, Mike Westby, Helen Bright

**Affiliations:** 1 Infectious Diseases Group, Pfizer Global Research and Development, Sandwich, Kent, CT13 9NJ, UK

## Abstract

**Background:**

The BALB/c mouse is commonly used to study RSV infection and disease. However, despite the many advantages of this well-characterised model, the inoculum is large, viral replication is restricted and only a very small amount of virus can be recovered from infected animals. A key question in this model is the fate of the administered virus. Is replication really being measured or is the model measuring the survival of the virus over time? To answer these questions we developed a highly sensitive strand-specific quantitative PCR (QPCR) able to accurately quantify the amount of RSV replication in the BALB/c mouse lung, allowing characterisation of RSV negative and positive strand RNA dynamics.

**Results:**

In the mouse lung, no increase in RSV genome was seen above the background of the original inoculum whilst only a limited transient increase (< 1 log) in positive strand, replicative intermediate (RI) RNA occurred. This RNA did however persist at detectable levels for 59 days post infection. As expected, ribavirin therapy reduced levels of infectious virus and RI RNA in the mouse lung. However, whilst Palivizumab therapy was also able to reduce levels of infectious virus, it failed to prevent production of intracellular RI RNA. A comparison of RSV RNA kinetics in human (A549) and mouse (KLN205) cell lines demonstrated that RSV replication was also severely delayed and impaired *in vitro *in the mouse cells.

**Conclusions:**

This is the first time that such a sensitive strand-specific QPCR technique has been to the RSV mouse system. We have accurately quantified the restricted and abortive nature of RSV replication in the mouse. Further *in vitro *studies in human and mouse cells suggest this restricted replication is due at least in part to species-specific host cell-viral interactions.

## Background

Respiratory Syncytial Virus (RSV) is the leading cause of lower respiratory tract infection (LRTI) in infants and children world-wide and is increasingly recognised as a cause of serious disease in adults and immune compromised transplant patients [1,2]. Over half of all children will be infected with RSV by their first birthday and by the age of 2 nearly all children will have been infected with RSV at least once [3]. LRTI caused by RSV infection is a major cause of both infant hospitalisation and infant viral induced death [4]. A number of medical treatments, including use of bronchodilators, palliative care (supportive ventilation, nitric oxide) and use of anti inflammatory agents are available but none of these treatments relieve the viral burden in RSV-infected patients. The only small molecule antiviral therapeutic agent for treating RSV is Virazole (aerosolised ribavirin), which has been shown to be of limited use because of its lengthy administration and questionable efficacy [5,6]. Palivizumab (Synagis) is a humanised monoclonal IgG1 antibody specifically directed to the RSV fusion protein which has been used prophylactically to good effect in at-risk infants. However, a therapeutic treatment did not result in significant clinical benefit [7]. Thus, there is a clear, unmet medical need to develop therapies able to ameliorate RSV disease [8-10].

RSV is a negative-stranded RNA virus belonging to the *Paramyxoviridae *family. The negative sense single-strand RSV genome comprises a RNA molecule encoding 11 proteins. Upon host cell infection positive-sense viral mRNAs are synthesised by the viral RNA polymerase, these mRNAs make use of host-cell machinery to synthesise viral proteins. Genome replication occurs via the production of a positive sense replicative intermediate (RI) RNA strand by the same viral RNA polymerase; this RI RNA is used as a template for the synthesis of more negative sense genome [11-13].

The use of *in vivo *models with good clinical translation is vital in the search for new treatments for disease A number of different animal models have been used to study RSV infection and replication and to evaluate potential therapies, including primates, bovines and rodents [14]. The majority of *in vivo *studies have been conducted using either the BALB/c mouse [15] or the cotton rat (*Sigmodon hispidus*) [16] models. The cotton rat is moderately permissive to human respiratory viral infection and RSV is able to replicate and produce viral progeny in the lungs [17]. The BALB/c mouse is also susceptible to RSV infection [18] and, though less permissive than the cotton rat [19], constitutes a more practical model due to the availability of a larger number of immunological and molecular reagents as well as the availability of transgenic animals. Like the cotton rat, the mouse requires inoculation with a high dose (usually 10^6 ^PFU) to achieve viral replication. The actual amount of viral replication occurring following infection with such a supra-physiological dose of RSV has never been accurately determined.

We therefore developed a strand-specific real-time quantitative polymerase chain reaction (QPCR) method to monitor the kinetics of RSV RNA replication in the mouse lung. BALB/c mice were infected with RSV A2 and viral RNA in mouse lungs were monitored over an extended time course. Levels of infectious virus in lungs were also measured. Taken together, results from these 2 assays showed that RSV RNA synthesis and viral replication was severely limited in the mouse. Treatment with a prophylactic antibody (palivizumab) did not affect viral RNA replication and persistence, but did impair the production of infectious progeny virus, indicating that abortive replication [16] occurs in the mouse. By contrast, positive sense viral RNA and infectious virus production were both disrupted by ribavirin. Further *in vitro *studies in human and mouse cells demonstrated that although both cell types were equally susceptible to infection; viral RNA synthesis was delayed and impaired in mouse cells. This finding suggests that a species-specific host-virus interaction inhibits the capacity for RSV replication in the mouse.

## Methods

### Animals

Female BALB/c mice (6-8 weeks old), specific pathogen free, were purchased from Charles River Laboratories and housed in an animal care facility in ventilated isolation cubicles. Water and chow were provided ad libitum. Mice were allowed to acclimate to the new environment for 1-2 weeks and housed in groups according to experimental setup. All experiments with animals were carried out in compliance with UK legislation and subject to local ethical review.

### Virus, cells and viral assays

RSV-A2 was obtained from Advanced Biotechnologies Inc. Stocks were produced by infecting Hep-2 cells at a multiplicity of infection (MOI) of 0.1 focus forming units (FFU) per cell. Following 4-5 days incubation, infected cells were harvested and snap frozen in dry ice and methanol and stored at -80°C. Viral titres were determined by a HEp2 based immunofluorescence assay and expressed as FFU/ml [20]. UV-inactivated RSV (UVRSV) was generated by exposing RSV A2 to UV radiation at 254 nm for 20 minutes using a Stratalinker (Stratagene). Loss of infectivity of UVRSV was confirmed by infecting Hep2 cells (MOI ranging from 0.1-1 FFU/cell). For animal studies, viral titres were expressed as geometric means +/- standard errors of means (SEM) for all animals in a group.

A549 cells (human lung carcinoma) and KLN205 cells (DBA/2 mouse lung squamous cell carcinoma) were purchased from ATCC and maintained in DMEM or EMEM respectively, each supplemented with 100 IU/ml of penicillin, 100 μg/ml streptomycin, 2 mM L-glutamine, and 10% foetal calf serum (FCS).

### Drugs

Ribavirin was obtained from Sigma-Aldrich and Palivizumab (Synagis, MedImmune) was obtained from Idis Ltd.

### RSV infection in vivo

For preliminary RSV replication and dynamics studies, mice were inoculated once intranasally (i.n.) with 50 μl of either RSV A2 (1 × 10^6 ^FFU per animal) or an equivalent concentration of UVRSV. One group of control mice was left untreated. Animals were sacrificed at 1, 5, 8, 17, 24, 48 and 72 hours and 7, 10, 37 and 59 days after infection (3 mice per time point). The lungs were removed from the thorax, dissected into two and each weighed. One lung was placed into RNAlater (Ambion) for subsequent RNA extraction and Taqman analyses. The second lung was processed by hand-held homogeniser (Omni) in 1 ml MEM (Invitrogen). Homogenates were centrifuged, clarified viral supernatant diluted 1:3 in MEM and 50 μl used in triplicate in immunofluorescence assay [20].

For experiments conducted to investigate inhibition of RSV replication, one group of animals were administered a single intramuscular injection of palivizumab (5 mg/kg of body weight) 24 hours prior to infection with RSV. A second group were administered ribavirin (100 mg/kg of body weight) intraperitoneally one hour prior to RSV challenge. These groups, plus a further untreated group, were inoculated intra nasally with 75 μL RSV A2 (2.6 × 10^6 ^FFU/mouse). Ribavirin treatment was re-administered 5 hours post virus inoculation and twice daily dosing of this compound continued for a further day. Ribavirin treatment was not administered on day 2. Dosing continued on day 3 at 50 mg/kg twice daily until day 6 post virus infection. Animals were sacrificed at 1, 8, 24, 48 and 72 hours and 5, 7 and 10 days post infection (6 animals per group per time point). Lungs were harvested for viral titrations and RNA extraction.

### In-vitro transcript standard production

A region of the RSV A2 nucleocapsid domain was isolated using a nested primer approach. RSV A2 viral RNA was prepared from crude preparation using the QIAamp viral RNA minikit (Qiagen). RNA was reverse transcribed using the High Capacity cDNA reverse-transcription kit (Applied Biosystems) with random primers. PCR was conducted using *Pwo *Superyield polymerase (Roche) with external primers (Table 1) at an annealing temperature of 60°C for 35 cycles followed by nested primer (Table 1) PCR using cycling conditions as described above. Gel-purified PCR product was restriction-cloned into pGEM-4Z vector (Promega), grown in Oneshot TOP10 chemically competent *E. coli *(Invitrogen) and plasmid purified by QIAprep Spin Miniprep Kit (Qiagen). Clones were sequence-checked at Lark Technologies, UK. The insert plus bacterial promoter vector sequences of verified clones was isolated by PCR using *Pwo *Superyield polymerase with M13 forward (-20) and reverse primers at an annealing temperature of 55°C for 35 cycles. Positive and negative sense in vitro transcripts were synthesised by Sp6 and T7 RNA polymerase (Promega) respectively, these products were treated with Turbo DNase (Ambion) and purified by 3 M sodium acetate (pH 5.5) precipitation. Stocks of 10^8 ^absolute copies per μl were prepared and stored at -80°C.

**Table 1:** Primer sequences used for RNA standard generation, cDNA synthesis and QPCR.

Primer	Sequence
In vitro standard external positive sense	TCCAGCAAATACACCATCCA

In vitro standard external negative sense	CTGCTTCACCACCCAATTTT

In vitro standard nested positive sense	ATA**GAATTC**GGTATGTTATATGCGATGTCTAGGT^1^

In vitro standard nested positive sense	ATA**GGATCC**TGCTAAGACTCCCCACCGTAA^2^

Positive sense RNA-specific cDNA synthesis	**CGGTCATGGTGGCGAATAA**TCCTGCAAAAATCCCTTCAACT^3^

Negative sense RNA-specific cDNA synthesis	**CGGTCATGGTGGCGAATAA**ACTTTATAGATGTTTTTGTTCA^3^

Positive sense-specific QPCR primer	CCCCACTTTATAGATGTTTTTGTTCA

Negative sense-specific QPCR primer	TCCTGCAAAAATCCCTTCAACT

QPCR tag primer	CGGTCATGGTGGCGAATAA

Probe	FAM-TTGGTATAGCACAATCTTCTACCAGAGGTGGC-TAMRA

^1 ^Sequence contains an EcoRI restriction site (bold, underlined)

^2 ^Sequence contains a BamHI restriction site (bold, underlined)

^3 ^Tag sequence is indicted (bold, underlined)

### Strand-specific real time QPCR

RNA was prepared from mouse lungs using an RNeasy kit (Qiagen) following manufacturer's instructions. First strand cDNA was synthesised from RNA using Reverse Transcription Reagents (Applied Biosystems) with gene specific primers targeted to the positive or negative sense RSV A2 nucleocapsid region RNA (Table 1). Primers contain a tag sequence recognised by a tag-specific primer in QPCR reactions; this reduces the detection of non-specific, self-primed cDNAs [21]. Reactions (10 μl) comprised 1 × reaction buffer, 5.5 mM MgCl_2_, 0.5 mM dNTP mix, 2.5 μM strand-specific primers, 4 U RNase inhibitor and 12.5 U reverse transcriptase with 4 μl total RNA preparation in water. Reactions were performed at 50°C for 40 mins followed by 95°C for 5 mins. Positive strand detection by QPCR was performed using TaqMan^® ^Universal PCR mastermix (Applied Biosystems) with positive sense RNA specific primer, 800 nM tag-specific primer and 100 nM probe (Table 1). Reactions were performed using an Applied Biosystems 7900 HT. Samples were held at 50°C for 2 mins followed by 95°C for 10 mins and then 40 cycles of 95°C for 15 secs and 60°C for 1 min. Negative sense strand detection was performed as described for the positive sense RNA reaction but substituting the positive sense RNA specific primer for a negative sense RNA primer. Positive and negative sense RNA transcript standard ranges (10-10^7 ^absolute copies/μl) were processed alongside samples. The limits of detection for this assay were defined as values measured outside the range of the standard curves. RSV copy number per μl of total mouse lung RNA were normalised to beta-actin detected using commercially available TaqMan^® ^VIC/MGB primer-limited endogenous control (Applied Biosystems) with random-primed 1^st ^strand cDNA synthesised using the High Capacity cDNA reverse-transcription kit (Applied Biosystems). Absolute values of normalised RSV copy number were subsequently divided by the weight of the lung tissue from which RNA was extracted and expressed as normalised copy number/g lung wt.

### RSV infection *in vitro*

To investigate whether RSV RNA synthesis occurs effectively in a mouse cell line compared to a human cell line in vitro, human lung carcinoma cells (A549) and mouse lung epithelial squamous cells (KLN205) were plated at a density of 1 × 10^4 ^cells per well in 96 well plates and infected with RSV A2 to yield various multiplicities of infection (MOIs) ranging from 1 × 10^-3 ^to 1. Media containing 10% FCS was replaced with fresh media containing 2% FCS after 24 hours. Cells were lysed with RLT buffer (Qiagen) at 1, 8, 24, 48 and 72 hours and after 5 (A549) or 6 (KLN205), 7 and 10 days. Total RNA was prepared using the RNeasy 96 kit (Qiagen). RSV strand-specific QPCR was performed as described above. RSV copy number per μl of total RNA were not normalised to beta-actin but rather analysed separately due to variable rates of cell death observed throughout the experiment and expressed as RSV copy number.

### Statistics

For QPCR analyses the ratio of positive to negative copy number is analysed on the logarithmic scale. Treatments are compared to untreated RSV infected controls at each time point by two-sample t-test incorporating Satterthwaite's adjustment to the degrees to freedom. To allow for testing of multiple time points within a treatment a Bonferroni adjustment was made to achieve an approximate 5% significance level within that treatment. Infectious virus assay data were analysed by 1 way analysis of variance (ANOVA) for significant differences (p = < 0.05) between treated groups and untreated RSV infected controls at each time point.

## Results

### Development of an RSV strand-specific real time quantitative PCR method

**Figure 1:**
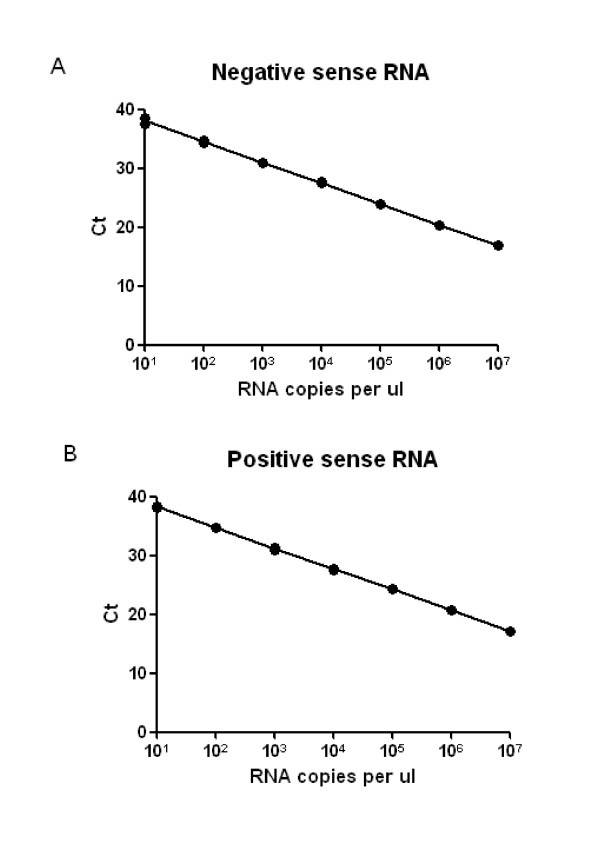
**An RSV strand-specific QPCR method**. Strand-specific priming was performed during cDNA synthesis and QPCR was performed using a primer/probe set designed to amplify part of the nucleocapsid region. A) Negative sense RNA standard curve. B) Positive sense strand RNA standard curve. Duplicate measurements are plotted.

A strand-specific QPCR method was developed to study RSV intracellular RNA dynamics. This method distinguishes between negative sense (genomic) RNA and positive sense RNAs (nucleocapsid mRNA and RI RNA) by discrimination at the 1^st ^strand cDNA synthesis stage. The strand-specific RSV primers used in the reverse transcription stage contain tag sequences that are incorporated into specifically primed cDNA and this sequence can be specifically targeted by a tag-specific primer during QPCR cycling (Table 1). The use of this tag is designed to reduce detection of cDNAs synthesised due to RNA self-priming in the reverse transcription reaction. Standard curves generated using in vitro transcribed RNA standards to monitor negative (Figure 1A) and positive (Figure 1B) strand specific QPCR revealed that both assays were ≥95% efficient with R^2 ^values above 0.99 (Table 2). The specificity of the reactions was assessed by spiking positive and negative sense RSV RNA standards into naïve mouse lung RNA and using both positive and negative strand-specific reagents to measure RSV RNA. The detection of non-specific RNA strand in mouse lung RNA background was <0.001% of specific strand detection in both positive and negative sense-specific reactions (Table 2). No signal was detected when naïve mouse lung alone was assayed, ruling out any non-specific effect from self-priming RNA species.

**Table 2:** Standard curve fits, R^2 ^and specificity of the QPCR.

RNA strand	Slope	Reaction Efficiency (%)	R^2^	Specificity (% non-specific strand detected)
Negative (genomic)	Y = -3.44x+41.11	95	0.997	< 0.001

Positive (mRNA and RI)	Y = -3.37x+41.06	98	0.997	< 0.001

Specificity was assessed by spiking non strand-specific RNA standards into naïve mouse lung RNA. Detection of spiked non strand-specific RNA is expressed as a percentage of measured specific sense standards spiked to mouse lung. Reaction Efficiency defined as 10^(-1/slope) ^- 1.

### RSV replication dynamics in BALB/c mouse lungs

RSV RNAs were analysed in the lungs of female BALB/c mice dosed i.n. with 10^6 ^FFU per animal over a 59 day period. Another group were infected with UV-inactivated RSV as a control.

**Figure 2:**
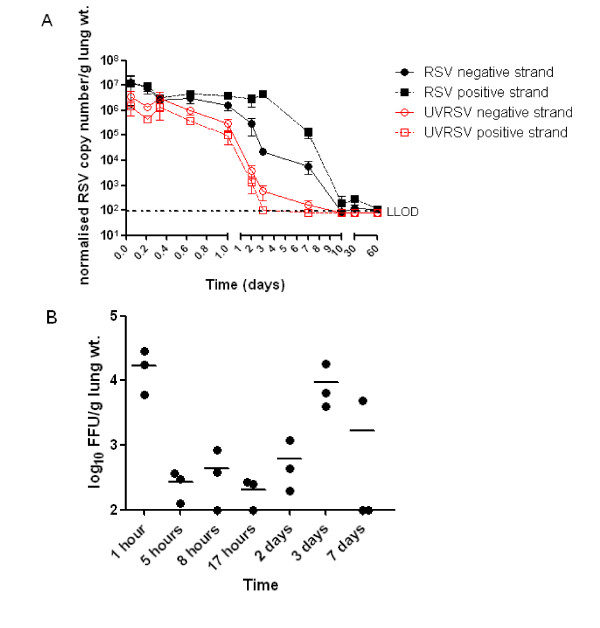
**RSV infection and replication in BALB/c mouse lungs**. Mice were dosed with either 1 × 10^6 ^FFU RSV A2 or an equivalent concentration of UV-inactivated RSV. Three mice per treatment were sampled at 1, 5, 8, 24, 48 and 72 hours and after 7, 10, 37 and 59 days post-infection. One lung per animal was processed for QPCR analyses, the other for infectivity assay. A) Levels of positive and negative sense RSV RNA in mouse lungs were monitored using strand-specific QPCR. Normalised RSV copy number was determined from strand-specific RNA standard curves corrected by beta actin arbitrary copy number. Means ± SEM for 3 animals per time point are plotted. Lower limit of detection = 80 actin normalised copies per gram lung wt. B) Infectious live virus in mouse lungs was monitored by FFU assay (lower limit of detection = 10^2 ^FFU/g lung wt.) up to day 7 post infection. Individual measurements are plotted and bars indicate mean values.

Mean normalised copy number/g lung wt. of both positive and negative RSV strands from infected mouse lung RNA preparations remained above 1 × 10^6 ^for 24 hours following infection (Figure 2A). From 24 hours onwards the amount of RSV negative strand reduced and this trend continued until day 10 when mean measured RNA reached a basal level of approximately 10^2 ^normalised copies/g lung wt. that persisted to 59 days post-infection. By contrast, the mean positive sense strand RNA remained above 10^6 ^normalised copies/g lung wt. until 72 hours post-infection. The mean negative and positive strand UVRSV RNA both declined in a time dependent manner from >10^6 ^normalised copies/g lung wt. 1 hour post infection and could not be detected after day 7 p.i.(Figure 2A). FFU assay performed on lung homogenates revealed a high mean FFU/g lung wt. of >10^4 ^at 1 hour post-dosing that was markedly reduced to <10^3 ^FFU/g lung wt. by 5 hours (Figure 2B). Infectious virus remained at this low level until 72 hours post infection when an increase to 10^4 ^FFU/g lung wt. was observed. Infectious virus reduced again on day 7. No infectious virus was detected from lungs excised from UVRSV dosed mice. Note that no RSV RNA or infectious virus could be detected in the lungs of control, untreated mice. These FFU data agree well with previously published results describing detection of infectious virus from RSV-infected BALB/c mouse lungs over a time-course [22].

### Effect of ribavirin and palivizumab on RSV replication in BALB/c mouse lungs

Having conducted a time-course overview of intracellular RSV RNA in BALB/c mouse lungs, we investigated the effects that palivizumab and ribavirin treatments have on RNAs in RSV-infected BALB/c mice and how these correlated with their effects on infectious virus production. A group of mice infected with 2.6 × 10^6 ^FFU RSV were treated prophylactically with palivizumab (5 mg/kg of body weight) 24 hours prior to infection with RSV. A second group were administered ribavirin (100 mg/kg of body weight) intraperitoneally one hour prior to RSV challenge and re-administered throughout the experiment as described in Materials and Methods. Untreated RSV infected mice were also monitored in this experiment.

**Figure 3:**
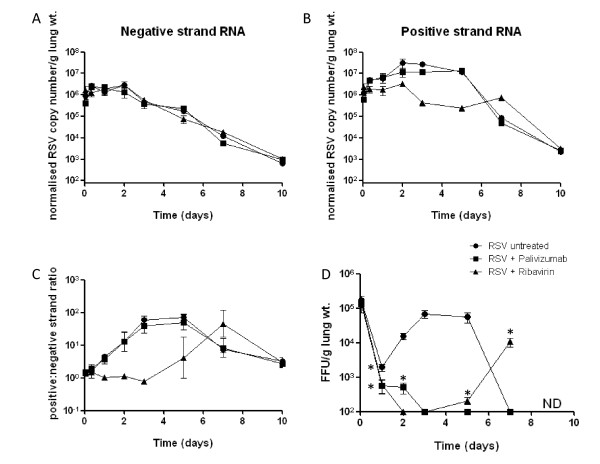
**Palivizumab and ribavirin reduce infectious virus in mouse lungs, but only ribavirin affects intracellular viral replication**. Naïve Balb/c mice and mice treated prophylactically with 5 mg/kg palivizumab were infected intra nasally with 2.3 × 10^6 ^FFU RSV A2. A third group were treated with ribavirin prior to RSV administration and throughout the study period as described in materials and methods. A) Negative and B) positive sense strand RSV RNAs were quantified by strand-specific QPCR. Means ± SEM for 6 animals per time point are plotted. Normalised RSV copy number was determined from strand-specific RNA standard curves corrected by beta actin arbitrary copy number. C) A ratio index of positive to negative strand RNA was constructed and time course profiles for ribavirin and palivizumab treatments are plotted against untreated RSV infected values. Means ± 95% confidence intervals (n = 6) are shown. D) Infectious live virus in mouse lungs was monitored by FFU assay throughout the time-course. Means ± SEM (n = 6) are plotted. Asterisks indicate significant difference (p ≤ 0.05) to untreated RSV-infected values at each sampling time. No data was collected post day 7 and is depicted as ND on the graph.

The use of either ribavirin or palivizumab had no effect on the quantities of intracellular negative sense genomic RNA measured throughout the experiment when compared to untreated RSV dosed mice (Figure 3A). However, ribavirin treatment did correlate with an alteration in the time course profile of positive sense RI RNA in mouse lungs compared to untreated RSV dosed mice (Figure 3B). There was a ≥1 log reduction in mean positive strand RNA relative to untreated RSV infected mice on days 3 and 5. There was no drop in positive strand copy numbers between days 5 and 7 in ribavirin treated mice, however positive strand copy numbers decreased to between 10^3^-10^4 ^normalised copies/g lung wt. on day 10, as was also measured in untreated mice. In palivizumab treated mice the positive sense RNA profile tracked closely that observed in untreated RSV infected mice. Measured RNA quantities were expressed as ratios of positive to negative strand RNA for each treatment (Figure 3C). Statistical analyses reveal that the positive/negative RNA ratio in ribavirin treated mouse lungs is significantly lower than that of untreated mice at days 1, 2, 3 and 5, and significantly higher at day 7. There is no significant difference to untreated RSV infected mice at day 10. It should be noted that dosing of ribavirin to mice was stopped at day 6, which coincides with the time at which the ratio of positive to negative strand RNA in ribavirin treated mouse lungs switched from being significantly lower than untreated RSV dosed mice at day 5 to significantly greater at day 7. The RSV RNA strand ratio from palivizumab-treated mouse lungs is not significantly different to that of untreated RSV infected mice at any time point (Figure 3C).

Infectious virus was quantified from lungs 1 hour post-infection by FFU assay (Figure 3D). Mean values from the 3 RSV infected groups were all approximately 10^5 ^FFU/g lung weight. In untreated RSV infected mice, levels of quantified infectious virus increased by 1-2 logs from approximately 10^3 ^FFU/g lung wt. at 24 hours to almost 10^5 ^FFU/g lung wt. at 3 days post infection. Measured infectious virus remained above 10^4 ^FFU/g lung weight to day 5 but became undetectable at 7 days. In ribavirin treated mice infectious virus in lung homogenates was significantly lower than in untreated RSV infected mice at 24 hours and was undetectable at 48 hours. Infectious virus was again detectable in this group at day 5 and increased at day 7. This increase coincides with a persistence of positive strand RNAs above 10^5 ^copies/g lung wt. at a time when positive sense RNA in untreated mice fell below 10^5 ^copies/g lung wt. (Figure 3B).

Infectious virus detected in lungs from mice treated with palivizumab was significantly lower than untreated RSV infected mice at 24 and 48 hours post infection. Infectious virus was undetectable from palivizumab treated mouse lungs at all time points past 48 hours.

### RSV RNA replication is severely impaired in mouse cells *in vitro*

**Figure 4:**
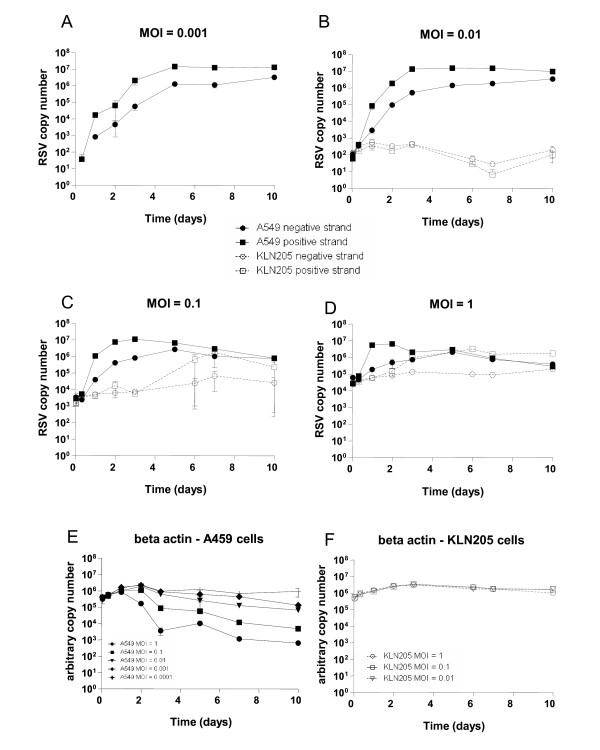
**RSV RNA synthesis in human and mouse cell lines**. A-D) Positive and negative sense viral RNA were monitored by strand-specific QPCR from A549 and KLN205 cells treated with RSV A2 at MOIs of A) 1 × 10^-3^, B) 1 × 10^-2 ^C) 1 × 10^-1 ^or D) 1. Copy numbers were determined from strand-specific RNA standard curves. RSV RNA was not detected in KLN205 cells treated with RSV at MOI = 1 × 10^-3^. E-F) Beta actin, expressed as arbitrary copy number, was measured by QPCR from E) A549 or F) KLN205 cells treated with RSV A2 at the MOIs shown. Means ± SEM (n = 2) are plotted.

In human A549 cells infected with a low MOI of 1 × 10^-3 ^or 1 × 10^-2^, viral RNAs increased from below the limit of detection at 1 hour post infection to maximum levels (negative sense >10^6 ^copies; positive strand >10^7 ^copies) at day 5 which were sustained up to the end of the experiment at day 10. When the cells were infected with higher MOIs of 1 × 10^-1 ^or greater, the positive and negative RSV RNA attained similar maximum levels to those observed in the lower MOI infections (negative sense >10^6 ^copies; positive strand >10^7 ^copies) (Figure 4C and 4D). Viral RNA reaches maximum expression values earlier in cells infected with higher MOI. A decrease in measured negative and positive RNA was observed in MOI 1 × 10^-1 ^and 1 infections after day 5 (Figure 4C and 4D) which correlated with a progressive decrease in beta actin gene expression, indicative of cell death (Figure 4E).

In mouse KLN205 cells infected with RSV at a low MOI of 1 × 10^-3 ^no viral RNA could be detected (Figure 4A). At an MOI of 1 × 10^-2 ^very low levels of positive and negative sense RSV RNA could be detected (Figure 4B), but at day 10 the mean amounts of neither strand were higher than those measured 1 hour post-infection (10^2 ^copies).

In cells infected at a MOI of 1 × 10^-1^, mean positive sense RSV RNA increased by 2 logs from 10^4 ^copies on day 3 to 10^6 ^copies by day 7 (Figure 4C). However, no increase in negative sense RNA copy number was observed over the 10 day culture period. A limited increase in positive sense RNA was also observed in cultures infected with an MOI of 1, rising from day 2 (10^5 ^copies) to day 7 (10^6 ^copies) and was maintained until the end of the study at day 10. Similar to cells infected with the MOI of 1 × 10^-1^, no increase in negative strand RNA was observed (Figure 4D). Beta actin levels in the mouse KLN205 cells fell less than 0.5 logs between days 2 and 10 indicating that no appreciable cell death had occurred throughout the study (Figure 4F).

## Discussion

We have developed a strand-specific QPCR method to measure RSV *in vitro *and *in vivo*. This method distinguishes between negative sense viral RNA (genome) and positive sense RNA (replicative intermediate and nucleocapsid mRNA). Using this method, we provide a detailed insight into RSV RNA production in infected BALB/c mouse lung. To our knowledge, this is the first time that a strand specific method has been applied to profile RSV RNA dynamics in the BALB/c mouse over such a detailed time course.

Early viral RNA synthesis in mouse lungs is characterised by absolute measures of positive and negative sense RNA being equivalent at infection, followed by a 1-2 logs relative increase in positive strand RNA by day 3 post infection. This disparity between RNA strands decreases again from day 7. It should be noted that this window of maximum disparity between the positive and negative strand copy numbers at day 3 coincides with the highest level of infectious progeny virus detected from mouse lungs following infection. It is known that paramyxovirus replicative intermediate RNA represent 10-40% of the genome [16], therefore the majority of positive strand RNA synthesis seen here is accounted for by nucleocapsid mRNA production.

That RSV genome and positive strand RNA can be detected in mouse lungs up to at least 59 days post-infection has been reported both here and elsewhere [15,23]. It therefore appears that mice are unable to fully clear the virus following infection. The fact that UV killed RSV was not detected by QPCR past day 7 supports this view of viral persistence. RSV persistence in the lungs has been reported from humans with chronic obstructive pulmonary disease (COPD) [24], although in another study, RSV infections in COPD were attributed to acute infection rather than low-level persistence [25]. The significance of persistent low levels of RSV in this and other conditions is unclear at present and further studies are required to elucidate the scope and impact of this phenomenon [26]. However, it is possible that low levels of persistent virus exist between RSV seasons and it is apparent that RSV persistence and strategies for complete viral clearance may be studied in rodent models.

Viral RNA replication has been studied by strand-discriminate QPCR previously in the cotton rat [16]. Viral genome levels increased by approximately 2 logs from 6 hours post infection to a peak measured on day 4 whereas our studies indicate that in the mouse lung total genomic RNA did not increase in this time frame. Indeed, in the mouse model we observed that viral genome load either decreased after 24 hours or (if a higher inoculum was applied), was maintained for a period of time before decreasing after day 5. These data suggest that RSV has a greater replicative capacity in the cotton rat model compared to the mouse. However until a direct head to head comparison is made between the two species, this cannot be concluded.

Ribavirin has been used extensively as an antiviral therapeutic. Its exact mode of action is poorly defined although several mechanisms have been proposed [27]. Here, as expected, ribavirin treatment had a marked effect on RSV intracellular RNA dynamics as evidenced by the reduction in positive sense RNA in mouse lungs. However, there was little difference seen in the time-course profiles of total genomic RNA in ribavirin treated and untreated RSV infected mice. This suggests that the amount of new genome synthesised following infection is only a small fraction of that dosed initially and that measuring positive sense RNA specifically is vital to the study of the intracellular viral processes in mouse lung following supra-physiologic dosing.

Prophylactic treatment of RSV-infected mice with the neutralising antibody palivizumab resulted in a reduction in infectious progeny virus detected in the lung, although a reduction in positive sense strand RNA was not observed. These findings agree with those previously observed in the cotton rat, where a lack of detectable progeny virus occurred despite intracellular replication taking place. This phenomenon was termed abortive replication [16]. The authors speculated that abortive replication could occur due to the blocking of production and release of large amounts of progeny virus despite infection occurring in the presence of high titres of neutralising antibody. Our data support this hypothesis. We conclude that the evaluation of antibody-mediated viral therapies in the mouse model may be confounded by the high viral titres required for effective infection.

To investigate whether the restricted replication pattern seen in the mouse is purely an *in vivo *phenomenon, we infected lung epithelial carcinoma cells from human (A549) and mouse (KLN205) with RSV and studied viral replication by strand-specific QPCR. One hour post infection, the input viral RNA levels were very similar in both human and mouse cells, irrespective of MOI or cell type, indicating that the mouse and human cells had been exposed to equivalent amounts of viral RNA. However, a clear increase in either viral RNA strand only occurred in mouse cells when they were infected with a high MOI of 0.1 or 1. This situation mirrors that which occurs in the mouse *in vivo *model in that an extremely high viral titre is required for replication [14]. Moreover, the increase in positive strand viral RNA was considerably delayed, occurring after a lag time of 3 days in culture suggesting that the virus has undergone a period of adaptation. Overall, RSV RNA synthesis in human A549 cells was at least 3 orders of magnitude more efficient than that observed in mouse cells, illustrating that RSV cannot replicate efficiently in mouse KLN205 cells. This data suggests that some host-specific block to viral replication exists, though a wider range of human and mouse cell lines require testing to confirm this.

It is unclear why the murine cells did not facilitate RSV RNA synthesis to the same extent as seen in human cells. It may be that RNA replication in KLN205 cells is inhibited either by the presence or absence of one or more host factors required for the viral life cycle. For example, it is known that RSV can modulate host cell anti-viral responses, such as the degradation of STAT2 by NS1 [28], which inhibits the interferon response. Poor replication of RSV in mouse embryo cells has been described previously [29]. This was attributed to the mouse interferon response as treatment of infected cells with anti-mouse interferon improved virus yields. Perhaps RSV is not able to modulate the mouse interferon response to the same extent as human interferon. Alternatively, it is also known that RSV requires host proteins to replicate efficiently. Phosphorylation of the RSV P protein by casein 2 is required for transcription elongation activity of the viral polymerase *in-vitro *[30]. It is plausible that species-specific differences in host factors may impair the ability of RSV to replicate efficiently in mouse cells, as is exemplified with HIV and APOBEC3G [31].

In conclusion, we have demonstrated and quantified the abortive and restricted nature of RSV RNA synthesis and replication in mouse using a highly sensitive and specific QPCR method. We have gone on to provide evidence that the impaired replication may be due to a murine host-virus interaction. We suggest a number of candidates and work is ongoing to identify these interactions.

## Competing interests

All authors are or were employed in a full-time capacity by Pfizer Research and Development.

## Authors' contributions

RB carried out the molecular and cellular studies and drafted the manuscript. DR carried out the *in vivo *and cellular assays and analysis and interpretation of data, EJM, MW and CL participated in the design of the study and analysis and interpretation of data. HB conceived of the study, participated in its design and coordination and helped to draft the manuscript. All authors read and approved the final manuscript.
